# CELL NON-AUTONOMOUS SEROTONIN SIGNALING MEDIATES STRESS RESISTANCE AND LONGEVITY

**DOI:** 10.1093/geroni/igz038.2676

**Published:** 2019-11-08

**Authors:** Scott Leiser, Hillary Miller, Shijiao Huang

**Affiliations:** University of Michigan, Ann Arbor, Michigan, United States

## Abstract

The ability of organisms to perceive and respond to their environment is crucial to their long-term survival. Recent studies in model organisms identify signaling pathways that perceive environmental stress and cell non-autonomously modify systemic physiology. These pathways often originate in the neurons, where key cells monitor the external environment for changes including food availability, air-quality, and the presence of dangerous toxins. Our previous work identified a key role for serotonin signaling in the induction of flavin-containing monooxygenase-2 (fmo-2) downstream of hypoxic signaling. fmo-2 expression is necessary and sufficient to promote stress resistance and longevity downstream of multiple genetic pathways, making it a useful tool for identifying key components of these pathways. Our current data defines environments, pathways, and signaling molecules that induce fmo-2 and subsequently increase lifespan. Our resulting data define key roles for serotonin signaling and fmo-2 that rely upon the perception of oxygen and food.

